# Vulnerable populations during COVID-19 response: Health-related quality of life among Chinese population and its influence due to socio-demographic factors and loneliness

**DOI:** 10.3389/fpubh.2022.857033

**Published:** 2022-08-15

**Authors:** Eliza Lai-Yi Wong, Jia Li, Shannon Yuen, Angel Hor-Yan Lai, Annie Wai-Ling Cheung, Peter Sen-Yung Yau, Eng-Kiong Yeoh

**Affiliations:** ^1^Centre for Health Systems and Policy Research, The Jockey Club School of Public Health and Primary Care, Faculty of Medicine, The Chinese University of Hong Kong, Hong Kong, Hong Kong SAR, China; ^2^Faculty of Social Sciences, Developmental and Educational Psychology, Leiden University, Leiden, Netherlands; ^3^Department of Applied Social Sciences, Faculty of Health and Social Sciences, The Hong Kong Polytechnic University, Hong Kong, Hong Kong SAR, China

**Keywords:** COVID-19 pandemic, health-related quality of life, loneliness, EQ-5D-5L, vulnerable populations

## Abstract

**Background:**

Infection control policy affected people's wellbeing during the COVID-19 pandemic, especially those vulnerable populations. This study aimed to compare the health-related quality of life (HRQoL) of the Hong Kong (HK) Chinese population under the pandemic with the normative profiles and explore its influencing factors, including socio-demographic characteristics, loneliness, and the interaction between them.

**Methods:**

A cross-sectional questionnaire survey (301 online and 202 in-person) was conducted between June and December 2020 among the adult Chinese population during the 2nd wave of COVID-19 in HK. HRQoL was measured by a Hong Kong validated EQ-5D-5L instrument (EQ-5D-5L HK). Loneliness was measured by a single-item question regarding the frequency of the participants reporting feeling lonely and their subjective social status was measured by the MacArthur Scale of Subjective Social Status. A series of Tobit regressions was conducted. The interaction terms between socio-demographics and loneliness were also examined to decide their association with HRQoL.

**Results:**

A total of 503 responses were collected. The level of HRQoL of the respondents was significantly lower than the referred norms profile among the local general population. The findings identified that younger age, single, a higher subjective social status, and a lower level of loneliness were significantly associated with better HRQoL. Moreover, age and marital status were significant moderators in the relationship between loneliness and HRQoL.

**Conclusion:**

The present study found that some population groups face additional vulnerabilities during the pandemic in terms of declined HRQoL. In addition, reducing loneliness can protect the HRQoL during the pandemic, especially among older people. This article provides useful information for policy-makers to design and promote effective services or provide education to improve the connection of people and recover from the global pandemic.

## Introduction

Initial cases of severe acute respiratory syndrome coronavirus 2 (SARS-CoV-2) were detected in Wuhan, China, in December 2019. Since then, ~219 million cases and 4.55 million deaths have been recorded worldwide. Social distancing measures have been launched by the governments to control the spread of the virus. For instance, the Government of the Hong Kong (HK) Special Administrative Region has promptly announced social distancing measures to limit the spread of the virus, such as mandatory wearing of face masks in all public areas, prohibiting large group gatherings, and restricting dine-in hours, closing all leisure facilities, and encouraging work at home.

While such strict measures proved to be effective in containing the spread of the virus, they have also had significant negative consequences on people's daily wellbeing. Health-related quality of life (HRQoL) is a multi-dimensional and interdisciplinary concept that has been used to reflect an individual's subjective evaluation of physical, mental, and social health (1). While there are a lot of studies investigating the HRQoL of COVID patients, survivors, and people with different health conditions specifically, a smaller number of studies have focused on the general population. As an exception, a Japanese study compared the HRQoL of the same cohort in 2017, 2020, and 2021 and found significant declines in HRQoL after the outbreak of the pandemic (2).

Populations with different socio-demographic characteristics may also face different levels of vulnerabilities with regard to the influence of the pandemic on their HRQoL. Some socio-demographic factors have been found to be significantly associated with a lower level of HRQoL, such as older age, female gender, unmarried, and lower socioeconomic status (3–6).

Loneliness is another established factor that can harm people's HRQoL (7, 8). Extensive evidence has suggested that levels of loneliness elevated during COVID-19 (9, 10). Indeed, a study in HK indicated that there were significant increases in loneliness among the older person with multi-morbidity after the onset of COVID-19 (11). However, few studies have examined the relationship between loneliness and self-reported health-related outcomes during COVID-19 specifically. According to studies conducted before COVID, loneliness can result in a decline in HRQoL. Recently, Liu *et al*. found that in the early stage of COVID, loneliness among young adults was significantly associated with lower self-reported mental health functioning, which is a domain of HRQoL (12).

As mentioned above, socio-demographic factors and loneliness may influence HRQoL during COVID-19, but their combining effects have received little attention. Studies conducted before the pandemic suggested that the influence of loneliness on people's HRQoL varies across socio-demographic characteristics. For instance, a study found that loneliness could predict decreased HRQoL after 3 years in women only (13). A meta-analytic study has also suggested that loneliness is associated with worse HRQoL among women than men (14). Furthermore, a high socio-economic status (SES) may act as a buffer against stress and attenuate the relationship between loneliness and health outcomes (15). On the contrary, those with a lower level of SES may have fewer resources to cope with loneliness, which will further influence their health outcomes. Therefore, some population groups may be even more vulnerable during the pandemic if loneliness is at a high level. Identifying the groups most at risk is critical for public health professionals to launch suitable interventions or programs to help them maintain health and wellbeing during this hard time.

The negative impacts of COVID-19 may differ across regions with different severity of the pandemic, infection control measures, and citizens' responses (16). HK, as one of the few regions that have managed to contain the spread of COVID-19 for a long time, is a unique context to examine the HRQoL of the population. While it is a good thing that HK citizens may have fewer concerns with the risks of being infected due to the relatively successful physical distancing measures, in the meantime, they may have experienced more inconvenience than those from regions with looser social distancing measures. How such a dilemma influences people's HRQoL needs to be investigated.

To address this knowledge gap, this study examined (1) the HRQoL of the HK general population during COVID-19 by EQ-5D-5L HK compared with its population norms obtained at the pre-pandemic period; (2) the effects of loneliness on HRQoL among the HK population during COVID-19; (3) the effects of socio-demographic factors on HRQoL among the HK population during COVID-19; (4) the interaction effects between different socio-demographic factors and loneliness. This study can help to provide additional evidence on how resources should be allocated among the vulnerable population groups, as well as provide insights on the formulation of suitable policies and responses to improving population health.

## Materials and methods

### Study design

A cross-sectional survey with a structural questionnaire was conducted between June and December 2020 (the second wave of COVID-19 in HK). There was a rise in case of number in July 2020 to around 100 per day, which reduced to single digits by the end of August. Another wave arrived at the end of November with dozens of cases per day. During this period, the Hong Kong (HK) government launched social distancing measures, such as restricting the number of people dining in restaurants and implementing mask mandates. Due to the social distancing measures and concerns regarding virus spread, most of the questionnaires were filled in *via* online platforms (*n* = 301), with the rest being administrated in person (*n* = 202). Convenience sampling was adopted with the help of NGO partners. Those who were HK residents, aged 18 years or above, and able to understand Chinese were eligible for the survey. An information sheet, including the details of the study, was available at the beginning of the survey. Electronic consent was obtained from the participants and the participants who agreed to join the study filled in the questionnaire on their own electronic devices or with the help of interviewers. Data collected were retrieved from an online platform, and the downloaded database was password protected. Ethical approval was obtained from the Survey and Behavioral Research Ethics Committee of the authors' University (Ref. no: SBRE-19-755). All participants were required to consent to participate after receiving an explanation of the purpose of this study and their rights during participation.

### Measurements

HRQoL was measured using the HK Chinese version of the EQ-5D-5L instrument (EQ-5D-5L HK) (17, 18). Developed by the EuroQoL Group, EQ-5D consists of five dimensions to measure HRQoL for clinical and economical assessment: mobility (MO), self-care (SC), usual activities (UA), pain/discomfort (PD), and anxiety/depression (AD). For each dimension, there were five response levels indicating the severity of the participant's problem, if any: (1) no problem, (2) slight problems, (3) moderate problems, (4) severe problems, and (5) extreme problems. The best health state can be represented by “11111” and the worst can be represented by “55555”. The different health states were converted into a utility index ranging from −1 to 1, where full health is anchored at 1, death at 0, and a negative value is obtained when the health state is worse than death, the higher score indicating better HRQoL. In HK, a hybrid model without a constant after feedback module was selected as the final model to derive utility decrements (17, 18). Thus, the size of the coefficients reflects the relative weight placed on different kinds of health problems. For example, if mobility scores level 3 but all other dimensions scored level 2, the utility index by subtracting the coefficients (mobility: 0.1823; self-care: 0.0867; usual activities: 0.0672; pain/discomfort: 0.0756; and anxiety/depression: 0.0801) from 1, giving 0.5081. The lowest possible estimated value for the health state 55555 was estimated to be −0.8637 for the HK general population. The first population norm profile of HRQoL for Chinese residents aged 18 years and above was derived based on a representative sample (1,014 respondents) in HK using the preference-based value set of EQ-5D-5L HK at the pre-pandemic period. Thus, this norm profile was used in this study as the reference for the comparison of HRQoL between pre- and during the COVID-19 pandemic (19).

Loneliness was measured by a single-item question regarding the frequency with which the participant reported feeling lonely (1 = never, 2 = rarely, 3 = occasional, 4 = often, 5 = always). The single-item loneliness measurement was adopted by large surveys, such as The English Longitudinal Study of Aging (ELSA) (20, 21). A five-point Likert scale to measure the frequency of loneliness has also been widely used in previous studies among the Chinese population (22, 23). Due to the potential underestimation of loneliness level resulting from stigma concern and following the practice adopted by previous studies, the score of loneliness was further categorized into two groups: not lonely (never or rarely felt lonely) vs. lonely (occasional, often, and always felt lonely) (24).

Demographic characteristics included age, gender (female vs. male), religion (yes vs. no), education (< middle school, middle school, >middle school), living arrangement (living alone vs. living with others), and marital status (single vs. married/cohabited vs. divorced/widowed/separated) were also collected. Subjective social status (SSS) was measured by the MacArthur Scale of Subjective Social Status (1 = lowest to 10 = highest), which is a visual instrument to capture participants' sense of social status on a social ladder (25). This scale has been previously adopted in several studies undertaken on the HK population (26, 27). To account for the potential influence of the pandemic situation on participants' HRQoL, we also controlled for the stability of the pandemic on the date of the questionnaire survey collected. During our data collection period (June to December 2020), the government has adjusted physical distancing measures multiple times for virus control. Due to the rising infection number at the beginning of July 2020, the government tightened physical distancing measures on July 11. It did not loosen them until September 11, when the local infection number reduced to a single digit per day. On November 14, 2020, foreseeing another infection wave, the government tightened the measures again (28–30). Therefore, based on the infection number and the strictness of social distancing measures introduced by the government, we categorized the date of questionnaire survey collection into “stable” (before July 11, 2020; between September 11 and November 14, 2020) and “unstable” (July 12 to September 10, 2020; after November 14, 2020).

### Statistical analysis

The index score of EQ-5D-5L was calculated using the established HK value set (17, 18), and the norms profile for the general HK adult population was used for comparing the differences in the COVID-19 pandemic (19). Descriptive summary statistics were estimated for the mean index score, and percentage of people reporting any problem on each EQ-5D-5L dimension. Due to the non-normally distribution of EQ-5D-5L index score, univariate analyses, including Mann-Whitney U-tests, Kruskal–Wallis tests, and Spearman's correlations, were conducted to investigate the differences between participants with different demographic characteristics. Mann-Whitney U-tests and Kruskal–Wallis tests were used to compare group differences of socio-demographic variables in HRQoL, while Spearman's correlations were applied to examine the association between continuous socio-demographic variables (age and subjective social status) and the EQ-5D-5L score.

To explore the factors influencing the EQ-5D-5L index score, a Tobit regression model was adopted, which was considered appropriate for analysis due to the censored nature and the skewed distribution of the index score. Studies have shown in the ceiling effect of EQ-5D-5L that most respondents will report a perfect health state according to this instrument, which cannot differentiate respondents reporting perfect health. Therefore, the data should be interpreted to be censored to 1 and will generate a biased coefficient with conventional linear regression models (19). In the current sample, 200/533 (37.52%) participants score the highest score of 1 in our sample. To make the sample more comparable with the profile of the general HK adult population, the sample data were weighted based on gender and age using the 2020 HK census data as a reference. The regression was conducted among the weighted sample to examine the estimates among a sample that is closer to the HK general population. The independent variables included socio-demographic characteristics (including age, gender, marital status, education, religiosity, living arrangement, and SSS) and loneliness. The format of the questionnaire (face-to-face vs. online) and the stability of the pandemic during the data collection period was controlled in the model. The interaction effects between each socio-demographic variable and loneliness were then examined by adding an interaction term into the regression model. To avoid multicollinearity, all the continuous variables in the interaction term were centered. Akaike information criterion (AIC) and the Bayesian information Criterion (BIC) were computed to indicate the model fit, with smaller values indicating good model fits (31). Data were then analyzed using R version 4.0.3. Any *P* <0.05 values were regarded as statistically significant.

While a Tobit regression model has been frequently adopted by many studies examining factors influencing EQ-5D-5L due to its advantage in dealing with censored data (19, 32), the estimates in the Tobit model may be biased if the assumption of normality is violated (33). To check the robustness of the Tobit estimates, based on suggestions from previous studies, we conducted another two types of regression models as a sensitivity analysis: (1) a two-part model and (2) a generalized linear model. A two-part model consists of a logistic regression model with the binary outcomes (full health vs. non-full health) as the dependent variable and an ordinary least squares (OLS) model with the scores of non-full health as the dependent variable (34, 35). Including two parts in the model can mitigate the ceiling effect of the EQ-5D-5L score (34, 35). Generalized linear models allow for non-normal distribution. Gamma distribution of the EQ-5D-5L scores in the current sample was detected based on the Cullen and Frey graph (36). Moreover, because GLM with Gamma distribution requires a positive value of the dependent variable, we transformed the EQ-5D-5L score to a disutility score (1-EQ-5D-5L) as the dependent variable, the same as previous studies did (37, 38).

## Results

### Demographics

A total of 503 valid responses among the general HK adult population were collected. The profile of the present sample has younger age and a larger proportion of females than the general HK population, with the age of the respondents ranging from 18 to 89, with an average of 42.0 (SD = 21.9) (2020 HK population: 44.8). A total of 74.2% of participants were females (2020 HK population: 54.4%), while 41.4% of the participants were younger than 24 years old (2020 HK population: 10.1%). A total of 57.3% of the participants were single and 9.2% of them were widowed, divorced, or separated. A total of 62.6% of participants obtained an education level higher than an associate degree and 57.5% of them were non-religious. Only 8.0% of participants reported living alone. On a scale of 1–10, the average score of subjective social status was 5.37 (SD = 1.60). A total of 43.5% of participants were categorized as “not lonely” with the rest being “lonely”. Details are shown in Table 1.

**Table 1:** Sample characteristics and EQ-5D-5L HK index score (*N* = 503).

**Variables**	**Sample characteristics**	**EQ-5D-5L HK index score**					**EQ-5D-5L HK index score by socio-demographics**	
	***N* (%)/mean ±SD**	**Mean ±SD**	**min**	**1st quartile**	**Median**	**3rd quartile**	**Unweighted *p*-value**	**Weighted *p*-value**
Age	42.0 ± 21.9	0.862 ± 0.209	–	–	–	–	0.000***^a^	0.002**^a^
**Marriage status**								
Married/co-living	169 (33.6%)	0.829 ± 0.252	−0.865	0.773	0.924	1.000	0.000***^b^	0.000***^b^
Single	288 (57.3%)	0.919 ± 0.112	−0.251	0.856	0.920	1.000		
Widowed/divorced/separated	46 (9.1%)	0.829 ± 0.201	0.139	0.784	0.924	0.931		
**Education level**								
< Middle school	22 (4.4%)	0.818 ± 0.187	0.444	0.664	0.922	1.000	0.036*^b^	0.064 ^b^
Middle school/diploma/advanced diploma	166 (33.0%)	0.847 ± 0.228	−0.865	0.815	0.924	1.000		
Associate degree/degree/master/doctoral	315 (62.6%)	0.902 ± 0.153	−0.625	0.844	0.920	1.000		
**Gender**								
Male	130 (25.8%)	0.851 ± 0.269	−0.865	0.844	0.920	1.000	0.354 ^c^	0.191 ^c^
Female	373 (74.2%)	0.891 ± 0.143	−0.251	0.844	0.924	1.000		
**Religion**								
No	289 (57.5%)	0.900 ± 0.151	−0.416	0.844	0.920	1.000	0.034*^c^	0.135 ^c^
Yes	214 (42.5%)	0.854 ± 0.220	−0.865	0.844	0.920	1.000		
**Living alone**								
No	40 (8.0%)	0.888 ± 0.170	−0.652	0.775	0.844	0.924	0.003**^c^	0.001**^c^
Yes	463 (92.0%)	0.788 ± 0.292	−0.865	0.844	0.920	1.000		
Subjective social status	5.37 ± 1.60	0.862 ± 0.209	–	–	–	–	0.001**^a^	0.000***^a^
**Loneliness**								
Not lonely	219 (43.5%)	0.901 ± 0.175	−0.652	0.860	0.924	1.000	0.000***^c^	0.000***^c^
Lonely	284 (56.5%)	0.864 ± 0.190	−0.865	0.844	0.920	1.000		
**Questionnaire format**								
Online	301 (59.8%)	0.890 ± 0.173	−0.652	0.844	0.920	1.000	0.901 ^c^	0.050 ^c^
Face-to-face	202 (40.2%)	0.866 ± 0.200	−0.865	0.844	0.924	1.000		
**Stability of the pandemic**								
Unstable	139 (27.6%)	0.843 ± 0.221	−0.652	0.830	0.920	1.000	0.000^***c^	0.004**^c^
Stable	364 (72.4%)	0.894 ± 0.167	−0.865	0.844	0.924	1.000		

*p < 0.05; **p < 0.01; ***p < 0.001.

^a^Spearman's Correlation.

^b^Kruskal-Wallis test.

^c^Mann-Whitney U-tests.

### HRQoL during COVID-19 pandemic

**Figure 1:**
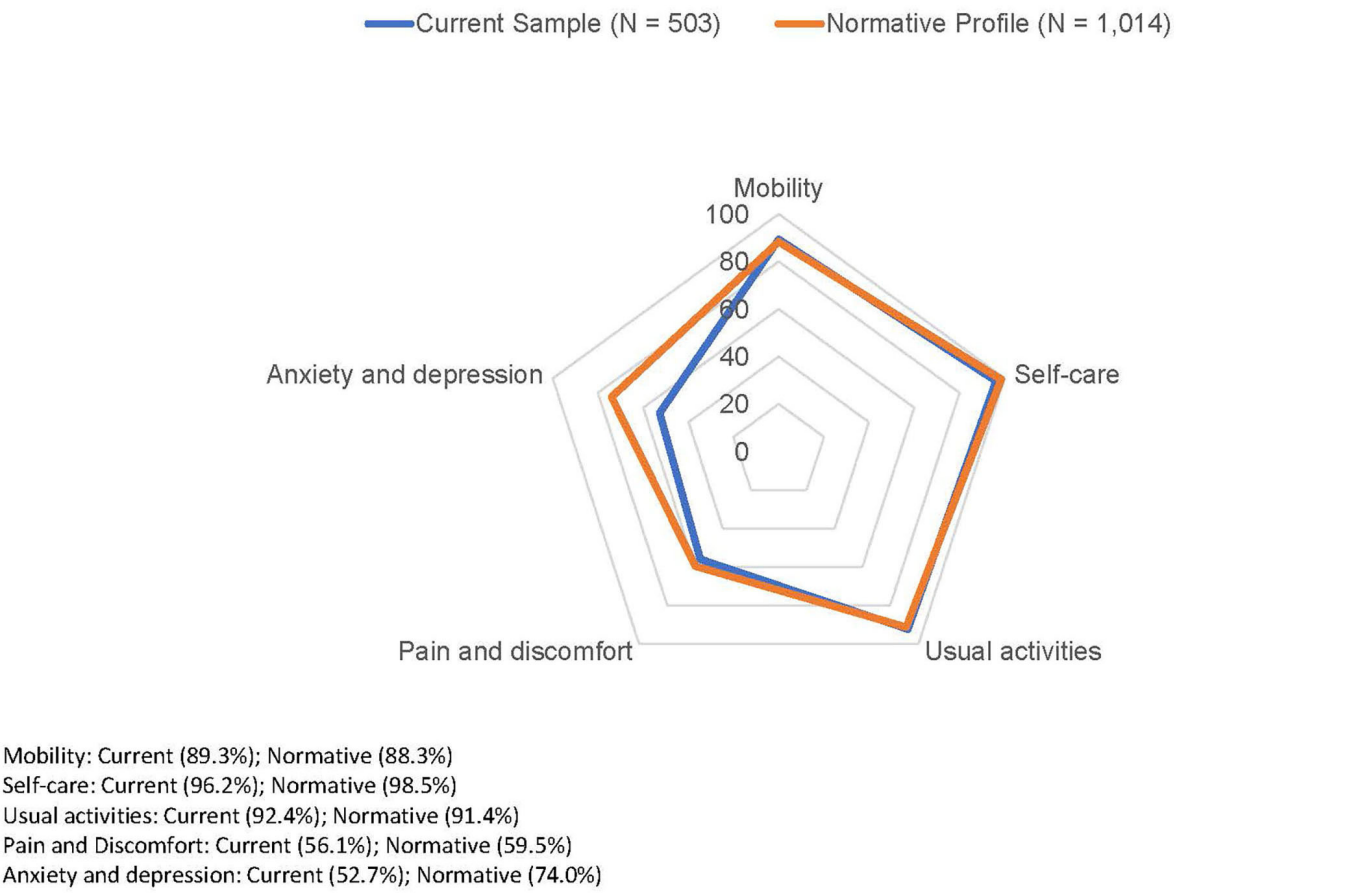
Percentages of participants reporting have no problem in five domains of EQ-5D-5L compared with HK normative profile.

The average EQ-5D-5L index score of the respondents was 0.862 (SD = 0.209), which was significantly lower than the norms profile (*M* = 0.915, SD = 0.128, *P* < 0.001) before the pandemic (19). Figure 1 shows the percentages of participants in the current sample and in the normative profile who reported having problems in the five domains of EQ-5D-5L. While 89.3, 96.2, and 92.5% of the participants reported no problems in mobility, self-care, and usual activities, only 56.1 and 52.7% reported no problems in pain/discomfort and anxiety/depression, respectively. In the normative profile, the most prevalently reported problem was pain/discomfort, while in the current sample, it was anxiety/depression that most participants reported having problems with. A total of 74.0% reported no problems with anxiety/depression, while the percentage was reduced to 52.9% in the current sample.

### Factors associated with EQ-5D-5L index scores

The EQ-5D-5L index scores by demographic information are also shown in Table 1. There were no significant differences in EQ-5D-5L index scores between the online and the in-person collected respondents (*P* = 0.050). Participants who participated in the survey during the stable months of the pandemic reported significantly higher scores in EQ-5D-5L (*P* = 0.004). In addition, after weighing the sample, there were no significant differences among participants of different gender, education, and religion (*P*s = 0.191; 0.164; 0.135). Participants who were single and living together with others reported significantly higher EQ-5D-5L index scores than those who were married/co-living or widowed/divorced/separated (*P* < 0.001), and solo-living (*P* = 0.001), respectively. A younger age (*P* = 0.002) and a higher SSS (*P* < 0.001) were also related to higher EQ-5D-5L indices. Participants in the “not lonely” group also reported a significantly higher EQ-5D-5L index score than those in the “lonely” group (*P* < 0.001).

As shown in Table 2, an older age was associated with lower EQ-5D-5L index score (*P* = 0.001). Single participants reported a 0.080-unit higher EQ-5D-5L index score than married/co-living participants [95% CI = (0.005, 0.156), *P* = 0.037]. One-unit increase in SSS was significantly associated with 0.028-unit increase in EQ-5D-5L index [95% CI = (0.012, 0.045), *P* < 0.001]. Lonely participants report a 0.152-unit lower EQ-5D-5L index score than lonely participants [95% CI = (−0.206, −0.098), *P* < 0.001]. The formats of the questionnaire and the stability of the pandemic had no significant associations with the EQ-5D-5L index score after controlling for all other variables.

**Table 2:** Tobit regression analyses of factors associated with EQ-5D-5L HK index scores.

**Variables**	**Estimate (B)**	**95% CI**		**Pr (>|z|)**
		**Lower limit (LL)**	**Upper limit (UL)**	
Age	−0.004**	−0.006	−0.002	0.001
Male (ref: female)	−0.037	−0.091	0.016	0.174
**Education**				
Middle school/diploma/advanced diploma (ref: < middle school)	0.020	−0.122	0.163	0.779
Associate degree/degree/master/doctoral (ref: < middle school)	0.034	−0.097	0.165	0.609
**Marriage status**				
Single (ref: married/co-living)	0.080*	0.005	0.156	0.037
Widowed/divorced/separated (ref: married/co-living)	0.027	−0.058	0.112	0.531
Religious (ref: non-religious)	−0.038	−0.088	0.013	0.148
Living with others (ref: living alone)	0.071	−0.022	0.163	0.136
Subjective social status	0.028***	0.012	0.045	0.001
Lonely (ref: not lonely)	−0.152***	−0.206	−0.098	0.000
Online survey (ref: face-to-face)	−0.046	−0.117	0.026	0.210
Pandemic (ref: stable)	−0.053	−0.117	0.012	0.108
AIC	306.636			
BIC	365.722			
Log likelihood	−139.317			

*p < 0.05; **p < 0.01; ***p < 0.001.

### Interactions between socio-demographic characteristics and loneliness

Interaction terms were also added to the regression model to examine the interaction between socio-demographic characteristics and loneliness in influencing the participants' EQ-5D-5L index scores (Table 3). Among all the socio-demographic characteristics, the interaction effects of loneliness with age or marital status on HRQoL were significant (as shown in Models 1 and 2, respectively). Adding interaction terms into the model improved the model fits (smaller values of AIC and BIC) compared to the baseline model to explain the impact on HRQoL.

**Table 3:** Tobit regression analyses of factors associated with EQ-5D-5L HK index scores with interaction terms.

**Variables**	**Model 1**				**Model 2**			
	**Estimate (B)**	**95% CI**		**Pr (>|z|)**	**Estimate (B)**	**95% CI**		**Pr (>|z|)**
		**LL**	**UL**			**LL**	**UL**	
Age	−0.002***	−0.005	0.001	0.179	−0.004***	−0.007	−0.002	0.000
Male (ref: female)	−0.037	−0.090	0.015	0.165	−0.038	−0.091	0.015	0.163
**Education**								
Middle school/diploma/advanced diploma (ref: < middle school)	0.023	−0.118	0.164	0.753	0.024	−0.118	0.165	0.743
Associate degree/degree/master/doctoral (ref: < middle school)	0.040	−0.089	0.170	0.543	0.039	−0.092	0.170	0.559
**Marital status**								
Single (ref: married/co-living)	0.081*	0.007	0.156	0.032	−0.005	−0.113	0.102	0.924
Widowed/divorced/separated (ref: married/co-living)	0.018	−0.066	0.102	0.670	0.059	−0.086	0.204	0.424
Religious (ref: non-religious)	−0.038	−0.088	0.012	0.136	−0.040	−0.091	0.010	0.120
Living with others (ref: living alone)	0.054	−0.039	0.146	0.256	0.066	−0.027	0.158	0.167
Subjective social status	0.028***	0.012	0.045	0.001	0.027**	0.010	0.044	0.002
Online survey (ref: face-to-face)	−0.045	−0.116	0.025	0.204	−0.042	−0.113	0.029	0.249
Pandemic	−0.047	−0.110	0.017	0.149	−0.049	−0.113	0.015	0.130
Lonely (ref: not lonely)	0.035	−0.118	0.188	0.655	−0.187	−0.255	−0.120	0.000
Age*lonely	−0.004*	−0.007	−0.001	0.011				
Marital status*lonely								
Single (ref: married/co-living)* lonely					0.119*	0.005	0.234	0.041
Widowed/divorced/separated (ref: married/co-living)*lonely					−0.039	−0.215	0.137	0.663
AIC	301.949				305.659			
BIC	365.257				373.188			
Log likelihood	−135.974				−136.829			

*p < 0.05, **p < 0.01, ***p < 0.001.

**Figure 2:**
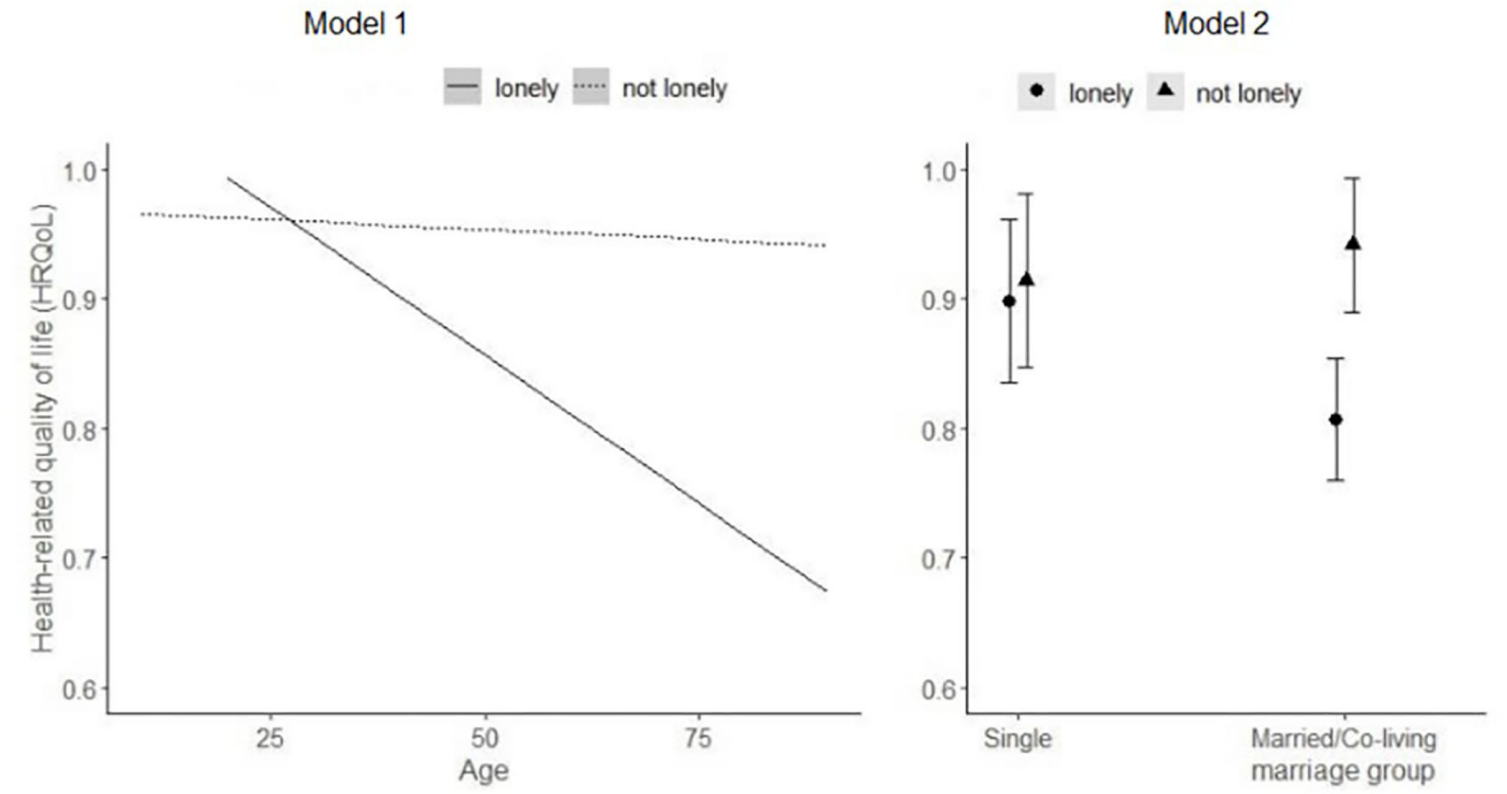
Interaction impact of loneliness with age or marital status on HRQOL under COVID-19.

The interaction impacts of loneliness with age or marital status on HRQOL are plotted in Figure 2 for ease of interpretation. In Model 1, the age differences in HRQoL differed by loneliness level. While both the younger and the older participants were at a similarly high level of loneliness, the age advantages of younger people in HRQoL were more apparent under the pandemic. When they both reported “not lonely”, older adults may not necessarily report a lower level of EQ-5D-5L score than younger people, indicating that older people may have some resilience in maintaining HRQoL. For the interaction effects between marital status and loneliness (Mode1 2), married or cohabited participants reported a lower level of HRQoL than single participants if they reported loneliness.

### Sensitivity analysis

We conducted two-part models and GLMs as sensitivity analysis, which showed similar findings to the Tobit models mentioned above. As shown in Tables 4, 5, in the logistic regression models of the two-parts model, being lonely was significantly associated with a lower likelihood of reporting full health. Participants who were single, lived together with others, and had a higher SSS were more likely to report full health. In the OLS models, age and marital status were significant moderators between loneliness and the utility score. Similar findings were also reported in the GLM models.

**Table 4:** Two-part models of factors associated with EQ-5D-5L HK index scores.

**Predictors**	**Logistic regression**				**OLS**			
	**OR (95% CI)**	***p***	**B (95% CI)**	***p***	**B (95% CI)**	***p***	**B(95% CI)**	***p***
Age	0.985 (0.965, 1.005)	0.137	−0.003** (−0.006, −0.001)	0.008	−0.006*** (−0.008, −0.003)	<0.001	−0.004** (−0.006, −0.001)	0.003
Male (ref: female)	1.179 (0.762, 1.823)	0.459	0.036 (−0.017, 0.088)	0.183	0.037 (−0.014, 0.088)	0.158	0.037 (−0.015, 0.090)	0.16
**Education**								
Middle school/diploma/advanced diploma (ref: < middle school)	1.094 (0.323, 4.027)	0.887	0.019 (−0.119, 0.157)	0.787	0.012 (−0.122, 0.146)	0.865	0.016 (−0.121, 0.153)	0.823
Associate degree/degree/master/doctoral (ref: < middle school)	1.225 (0.393, 4.230)	0.734	0.015 (−0.111, 0.141)	0.814	0.017 (−0.105, 0.140)	0.784	0.015 (−0.111, 0.140)	0.82
**Marital status**								
Single (ref: married/co-living)	1.962* (1.509, 3.670)	0.033	0.024 (−0.061, 0.110)	0.576	0.039 (−0.045, 0.123)	0.361	−0.087 (−0.214, 0.041)	0.183
Widowed/divorced/separated (ref: married/co-living)	0.731 (0.319, 1.592)	0.442	0.045 (−0.036, 0.126)	0.276	0.031 (−0.048, 0.110)	0.444	0.054 (−0.096, 0.204)	0.480
Religious (ref: non-religious)	0.705 (0.462, 1.072)	0.103	−0.004 (−0.055, 0.046)	0.865	−0.01 (−0.059, 0.040)	0.699	−0.009 (−0.059, 0.042)	0.74
Living with others (ref: living alone)	2.727* (1.096, 7.626)	0.04	0.013 (−0.071, 0.097)	0.759	−0.007 (−0.090, 0.075)	0.858	0.009 (−0.075, 0.093)	0.829
Subjective social status	1.297*** (1.126, 1.502)	<0.001	0.012 (−0.005, 0.029)	0.157	0.011 (−0.005, 0.027)	0.176	0.010 (−0.007, 0.027)	0.241
Lonely (ref: not lonely)	0.403*** (0.263, 0.614)	<0.001	−0.100*** (−0.154, – 0.045)	<0.001	0.213** (0.058, 0.367)	0.007	−0.143*** (−0.213, 0.074)	<0.001
Online survey (ref: face-to-face)	0.603 (0.345, 1.047)	0.073	−0.001 (−0.074, 0.071)	0.973	0.004 (−0.066, 0.075)	0.91	0.006 (−0.066, 0.078)	0.871
Pandemic	0.649 (0.384, 1.093)	0.104	−0.021 (−0.081, 0.039)	0.495	−0.013 (−0.072, 0.046)	0.659	−0.017 (−0.077, 0.044)	0.589
Age*lonely					−0.006*** (−0.009, −0.003)	<0.001		
Marital status*lonely								
Single (ref: married/co-living)*lonely							0.144* (0.019, 0.270)	0.024
Widowed/divorced/separated (ref: married/co-living)*lonely							−0.007 (−0.180, 0.165)	0.933
Observations	503		318		318		318	
R2 Tjur	0.112		0.118/0.083		0.167/0.131		0.133/0.093	
AIC	508.625		125.265		109.108		123.719	

*p < 0.05, **p < 0.01, ***p < 0.001.

**Table 5:** Generalized linear models of factors associated with EQ-5D-5L HK index scores.

**Predictors**	**Generalized linear models (disutility score)**					
	**B (95% CI)**	***p***	**B (95% CI)**	***p***	**B (95% CI)**	***p***
Age	0.003** (0.001, 0.004)	0.002	0.000 (−0.001, 0.002)	0.641	0.003** (0.001, 0.004)	0.001
Male (ref: female)	0.027 (−0.009, 0.064)	0.141	0.027 (−0.008, 0.063)	0.134	0.028 (−0.008, 0.065)	0.124
**Education**						
Middle school/diploma/advanced diploma (ref: < middle school)	−0.014 (−0.114, 0.087)	0.791	−0.019 (−0.117, 0.080)	0.711	−0.019 (−0.118, 0.080)	0.704
Associate degree/degree/master/doctoral (ref: < middle school)	−0.019 (−0.112, 0.073)	0.682	−0.028 (−0.119, 0.063)	0.545	−0.024 (−0.116, 0.069)	0.615
**Marital status**						
Single (ref: married/co-living)	−0.045 (−0.097, 0.006)	0.085	−0.045 (−0.096, 0.005)	0.080	0.030 (−0.040, 0.100)	0.398
Widowed/divorced/separated (ref: married/co-living)	−0.021 (−0.080, 0.039)	0.494	−0.011 (−0.069, 0.047)	0.707	−0.037 (−0.132, 0.057)	0.439
Religious (ref: non-religious)	0.023 (−0.012, 0.058)	0.191	0.023 (−0.011, 0.058)	0.180	0.025 (−0.009, 0.060)	0.152
Living with others (ref: living alone)	−0.037 (−0.103, 0.029)	0.268	−0.016 (−0.082, 0.049)	0.623	−0.034 (−0.100, 0.032)	0.311
Subjective social status	−0.013* (−0.025, −0.002)	0.022	−0.014* (−0.025, −0.003)	0.016	−0.013* (−0.024, −0.001)	0.028
Lonely (ref: not lonely)	0.094*** (0.058, 0.130)	<0.001	−0.112* (−0.211, −0.012)	0.029	0.133*** (0.087, 0.180)	<0.001
Online survey (ref: face-to-face)	0.012 (−0.036, 0.060)	0.629	0.014 (−0.034, 0.061)	0.573	0.010 (−0.038, 0.058)	0.676
Pandemic	0.038 (−0.006, 0.082)	0.088	0.031 (−0.012, 0.074)	0.158	0.035 (−0.009, 0.078)	0.120
Age*lonely			0.004*** (0.002, 0.006)	<0.001		
Marital status*lonely						
Single (ref: married/co-living)*lonely					−0.115** (−0.191, −0.040)	0.003
Widowed/divorced/separated (ref: married/co-living)*lonely					−0.016 (−0.103, 0.136)	0.787
Observations	503		503		503	
AIC	−206.653		−222.927		−212.601	

*p < 0.05, **p < 0.01, ***p < 0.001.

## Discussion

The present study examined the shift in HRQoL of the general HK adult population during the COVID-19 pandemic using the local validated EQ-5D-5L HK instrument. The EQ-5D-5L index score of the respondents was significantly lower than the referred population norms profile in HK. However, problems related to anxiety/depression were found to be more prevalent during the pandemic than pre-pandemic. The findings revealed that people with a higher level of loneliness tended to report lower HRQoL. In terms of the effect of socio-demographic factors on HRQoL, the findings indicated that people who were single or had a higher level of subjective social status tended to report higher HRQoL.

The COVID-19 pandemic has posed extra challenges to the personal, social, and professional lives of people of all ages. It also disturbed people's regular daily routines, such as attending community activities, seeking medical consultation, and meeting with families and friends, which will have detrimental effects on their physical and mental health. The significant decline of EQ-5D-5L index scores during the pandemic from the 2016 HK norm is consistent with some studies from other countries that the HRQoL of the general population was lower than the norms, such as in Portugal (39) during COVID-19. However, it was inconsistent with a previous study conducted in mainland China, which reported that the HRQoL of people in the examined city did not change much during the pandemic (5). Another study in Vietnam also did not find a significant difference between the pandemic EQ-5D-5L index and the normal scores (40). Such inconsistencies may be due to the different conditions of COVID-19 in different cities (5).

### Socio-demographics and HRQoL

Older age was significantly associated with a lower EQ-5D-5L index score after controlling of other variables. The findings are consistent with previous studies showing age as a risk factor of decreased HRQoL during COVID-19 (5). Possible explanations include that older people are more frequent users of the public healthcare system, community services, and other formal and informal caregiving. Such services have been greatly disrupted during the pandemic, which may influence their physical and mental health.

Subjective social status is a protective factor of HRQoL during COVID-19. This is consistent with previous research about the impacts of social positioning on one's health (41, 42). COVID-19 has accentuated the correlation between social disparity and health disparity (43, 44). People with higher socioeconomic status may experience less financial pressure due to the disruption of economic activities amid COVID-19. On the other hand, they have more resilience in conducting activities beneficial for their health under the condition of social distancing. A qualitative study previously undertaken among a local population in HK (45) suggested that socially disadvantaged people have suffered more from the economic and financial impacts of the COVID-19 pandemic; and they tend to have limited access to personal protective equipment, such as face masks, hand rub, and other disinfecting products. Work-from-home suggestions from the government also hardly applied to groups, whose jobs were usually unable to be completed at home. Poor housing conditions also exacerbated their vulnerability to disease infection due to overcrowding of families.

It is a bit surprising that compared to married/co-living couples, single participants were more likely to report a higher level of HRQoL and better health status on mobility and pain/discomfort. Tentative explanations include that continuous home office has increased the possibility of interpersonal conflict. Based on the vulnerability-stress-adaption model, Pietromonaco & Overall suggested that COVID-19 caused external stress and may result in harmful dyadic processes and decreased quality of romantic relationship (46). Another study adopting a nationally representative sample of American adults also revealed escalated partner conflicts during COVID-19 (47). Similarly, a study in mainland China has found that married participants reported a greater decline in emotional wellbeing during COVID-19 than their non-married counterparts (48). For those married, COVID-19 may have caused some family separation that may pose more adverse effects on married couples than single persons.

### Loneliness and HRQoL

The findings also revealed that loneliness was a strong predictor of HRQoL. Loneliness indicates a self-perceived insufficiency of social and emotional support. Loneliness, as a source of chronic stress, may cause pathologic hypervigilance and a dysfunctional immune system, which can further harm one's physical and mental health (49). Our finding is consistent with well-established empirical evidence indicating the concurrence of loneliness and a variety of health problems, such as depression, anxiety, cardiovascular diseases, hypertension, and chronic pain (7, 49–51).

### Interaction of socio-demographics and loneliness

The negative association between loneliness and HRQoL was more apparent among older people. It also indicates that reducing loneliness may be more rewarding for the HRQoL of older people in particular. At a lower level of loneliness, younger people's advantages in HRQoL decreased, which is consistent with previous literature regarding older people's resilience and favorable emotional regulation under adverse situations. It aligns with the socio-emotional selectivity theory (SST), indicating that older people tend to spend more energy on positive experiences rather than negative ones (52); while younger people tend to have more social and outdoor lives than other older age groups, the detrimental effects of social distancing may hit them more strongly than the older people. Indeed, it is common for younger people to report more mental health problems than the older population. A recent study conducted among American adults aged 18–76 found that the age advantages were sustained during COVID-19 (52). Such findings were also seen among samples from other countries, such as Canada (53). Other studies have also confirmed the greater risk of younger people suffering from mental health issues (54, 55); a global online survey collected data from 63 countries in the world and also found that younger people reported more mental health problems, including stress, depression, and anxiety, than middle-aged and older-aged population during COVID-19 (55). Marital status and loneliness also interacted with each other to influence HRQoL. Being married but at the same time feeling lonely may indicate separation or conflicts between partners, which will negatively influence their HRQoL.

### Limitation

This study possesses a few limitations. First, the data were cross-sectional data, and therefore no causal relationship can be drawn. Future studies should adopt a longitudinal or experimental approach to examine the factors influencing people's HRQoL. Moreover, the sample may be biased and not generalizable to the whole population. For instance, more than half of the participants were those who had access to the Internet for the online survey. Although we have controlled the collection in the regression, there may still be unobserved bias related to social desirability in the face-to-face samples. The sample was collected over a 6-months period, and the fluctuations in the COVID-19 situation may influence the respondents' responses to the questions. Second, it is noteworthy that the norms scores were generated in 2016. The differences between the scores collected in this study and the norms may be due to COVID-19, but it is also possible that the lower score is due to the continuous effects of the social and political unrest in HK (56, 57). Third, we used a single-item question to measure loneliness, which may not be able to capture different dimensions of loneliness, namely social and emotional loneliness. Fourth, this study did not measure the potential mediators between the socio-demographics, loneliness, and HRQoL, so that we cannot detect potential mechanisms between these variables. Fifth, the study sample was generated based on the non-institutional HK population, excluding those older adults who lived in residential care facilities. The care home residents may feel more alone and have poorer HRQoL due to the COVID-19 pandemic.

### Implications

The present study offers some implications for practice and policies during the COVID-19 pandemic. While COVID-19 has widespread negative effects on the health status of all populations, it also has the potential to intensify the social and health disparities. Although HK has so far succeeded in controlling the spread of the virus, the government should put more resources toward safeguarding socially disadvantaged groups who have fewer resources themselves to mitigate the adverse effects of COVID-19 in their careers, everyday lives, and health management.

Loneliness is another important issue because of the ongoing global pandemic and related social distance measures. While there are increasing instances of both family separation and considerably reduced amounts of friends and family gatherings, more mental health supports could be provided *via* phone, video-conferencing, or other telehealth channels. For instance, while long-term care facilities have been prohibited from visiting due to pandemic restrictions, the loneliness level of long-term care residents should be explored regarding their social needs and mental health. It is noteworthy that older people may not be the only group suffering from loneliness, and the younger adults cannot be assumed to experience a better condition only due to their skills in navigating online. All age groups are exposed to the risks of experiencing loneliness, with consequential outcomes in health and wellbeing. Appropriate and timely services or education should be developed and rolled out to meet the specific needs of people of all age groups for the connection.

## Conclusion

This study is among the first to report health-related quality of life (HRQoL) in the HK general population and identified several factors associated with HRQoL, including both demographic and psychosocial characteristics—loneliness during the COVID-19 pandemic, using the local validated EQ-5D-5L (EQ-5D-5L HK) instrument. It calls for future studies to look into the challenges faced and resilience needed by people from different backgrounds to cope with a wide range of difficulties during COVID-19. It also sets out potential implications for practitioners and policymakers to provide effective support or services and related education to help people recover from the global pandemic.

## Data availability statement

The dataset is available from the corresponding author on reasonable request.

## Ethics statement

The studies involving human participants were reviewed and approved by Survey and Behavioural Research Ethics Committee of the Chinese University of Hong Kong. The patients/participants provided their written informed consent to participate in this study.

## Author contributions

ELYW and EKY conceived the study design. JL and PSYY performed the analysis. JL, SY, and AWLC prepared a preliminary draft of the manuscript. All authors discussed the results and contributed to the final manuscript.

## Funding

The study was supported by The Hong Kong Jockey Club Charities Trust (2017/0096/F).

## Conflict of interest

The authors declare that the research was conducted in the absence of any commercial or financial relationships that could be construed as a potential conflict of interest.

## Publisher's note

All claims expressed in this article are solely those of the authors and do not necessarily represent those of their affiliated organizations, or those of the publisher, the editors and the reviewers. Any product that may be evaluated in this article, or claim that may be made by its manufacturer, is not guaranteed or endorsed by the publisher.
